# Torsion of wandering spleen involving the pancreatic tail

**DOI:** 10.1016/j.amsu.2019.12.001

**Published:** 2019-12-20

**Authors:** Francesco Colombo, Pierluigi D'Amore, Michele Crespi, Gianluca Sampietro, Diego Foschi

**Affiliations:** Department of Biomedical and Clinical Sciences, L. Sacco Hospital University of Milan, Italy

**Keywords:** Wandering spleen, Displaced spleen, Ectopic spleen, Splenic ptosis, Pelvic spleen, Splenectomy

## Abstract

**Background:**

Wandering spleen (WS) is a rare clinical entity resulting from the absence or maldevelopment of the ligaments normally involved in the attachment of the spleen in its normal position. WS can be a cause of acute abdomen leading to different complications ranging from torsion of the vascular pedicle to spleen infarction. Often, in absence of symptoms, it is an occasional finding during radiological exams and surgery represents the gold standard in the management of this unusual condition.

**Case presentation:**

We present a case of wandering spleen in a young nulliparous female with an history of recurrent abdominal pain. A preoperative CT-scan of the abdomen showed the presence of a multi-infarcted spleen twisted several times around its vascular pedicle, involving the tail of pancreas. The patient was electively treated with laparoscopic splenectomy.

**Conclusions:**

A laparoscopic approach is feasible in the treatment of this pathology. A correct and timely diagnosis of this condition is crucial to allow an organ preserving surgery. There are only few reported cases in literature describing an involvement of the tail of the pancreas in the torsion of the vascular pedicle. Complete excision of the ectasic veins tributaries of the splenic vein avoids the risk of postoperative vein thrombosis and bleeding.

## Highlights

• Wandering spleen is a rare clinical entity resulting from the absence or maldevelopment of the ligaments normally involved in the attachment of the spleen.
• CT-scan showed a multi-infarcted spleen twisted several times around its vascular pedicle, involving the tail of pancreas.
• Laparoscopic splenopexy or splenectomy are the gold standard for the treatment of wandering spleen.

## 1 Introduction

Wandering spleen (WS) is a rare clinical condition defined as an ectopic spleen moved from its normal anatomical position in the left hypochondrium because of the absence or malformations of normal splenic suspensory ligaments.

In this condition the spleen is attached to the hilum only by a long vascular pedicle and the organ “wanders” in the lower abdomen or in the pelvis; it results in hypermobility of the spleen that predispose to complications like torsion of the splenic pedicle, partial or complete infarction of the spleen associated with splenic vein thrombosis [1].

Its etiology has been a matter of debate; the congenital form occurs most probably as a result of a congenital incomplete fusion of the dorsal mesogastrium leading to the absence or the underdevelopment of the splenic ligaments [2]. Instead, the acquired form has been related to hormonal changes, multiparity, splenomegaly malaria, Hodgkin's disease and Gaucher's disease [3].

WS is characterized by a bimodal incidence as it generally manifests below one year of age and in the third decade of life affecting more frequently females in reproductive ages; anyway the global incidence is less than 0.2% [4].

Its clinical characteristics are variable, the patient often being asymptomatic or complaining with chronic abdominal pain. The acute presentation with sudden onset of sharp abdominal pain is usually due to torsion of the splenic pedicle [5]. Diagnosis is often an occasional finding during ultrasound exams or CT-scan of the abdomen performed for other reasons.

Operative treatment is nowadays the only recommended because of high complication rate of conservative treatment, including acute torsion of the vascular pedicle causing splenic gangrene, splenic abscesses, acute pancreatitis, necrosis of pancreatic tail secondary to torsion and upper gastrointestinal bleeding from gastroesophageal varices [6]. In case of WS with no complications, the first line treatment is elective laparoscopic splenopexis, performed in different techniques, including or not the use of a mesh [7,8]. Elective splenectomy for complicated WS has clear advantages over emergency surgery in terms of patient safety, and is generally recommended when the spleen parenchyma is too damaged to recover from several ischemic events, or for the spleen dimension [9]. An early diagnosis of WS is the only chance to allow organ preserving surgery [10].

This case has been reported in line with the SCARE criteria.

## 2 Case presentation

We report a case of a 18-year-old caucasian nulliparous female who referred to our attention in 2018 because of recurrent episodes of abdominal pain in known history of wandering spleen, casually diagnosed two years before after a gynecological routine visit.

She used to complain with frequent episodes of lower abdominal pain that caused multiple visits to the Emergency Department. The patient had never experienced any abdominal trauma and never undergone abdominal surgery.

**Fig. 1:**
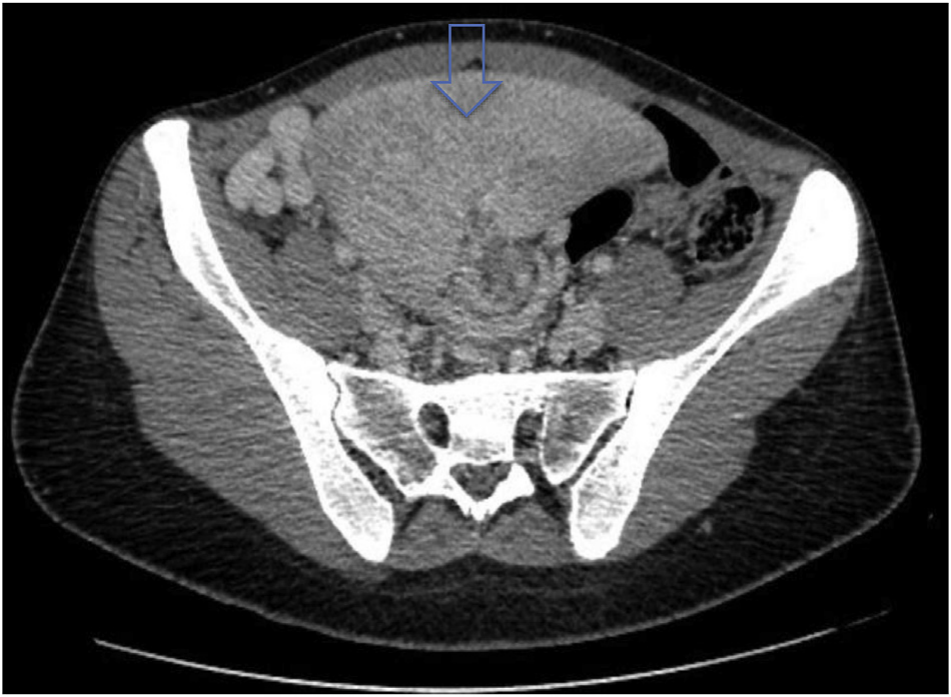
Contrast-enhanced axial CT image demonstrates the spleen in its pelvic position and the so called “whirl sign” of the splenic pedicle.

**Fig. 2:**
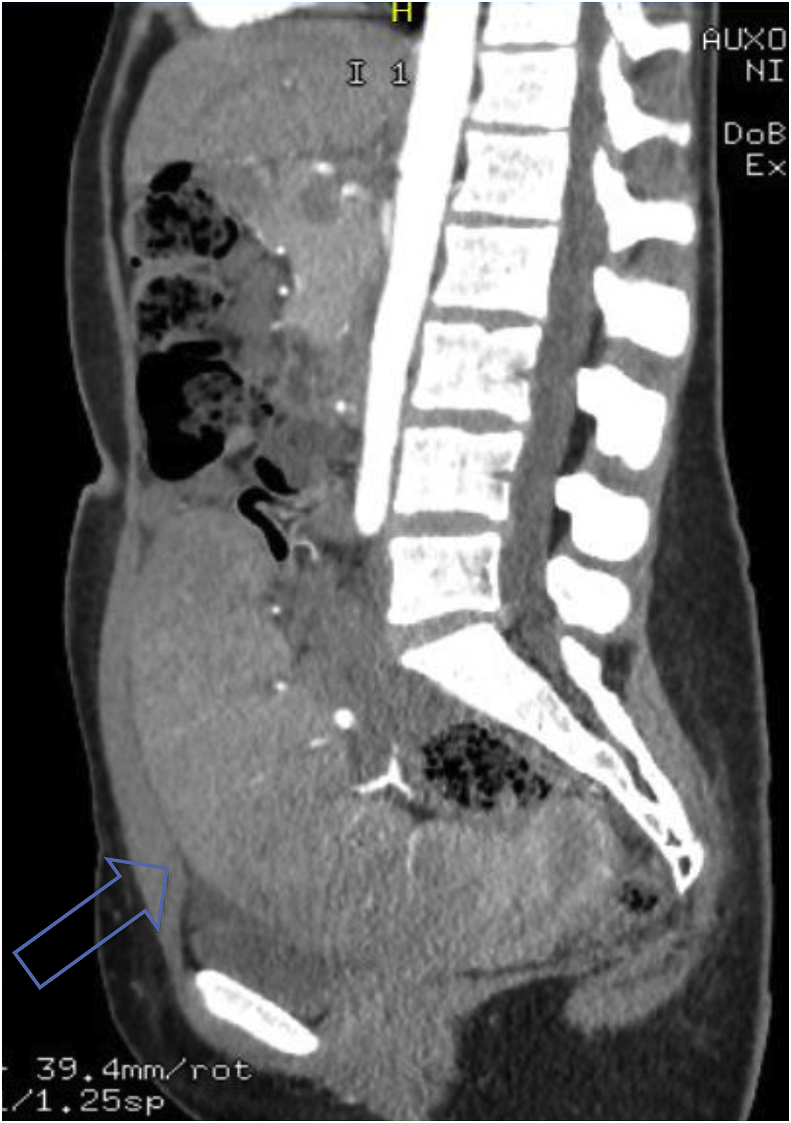
Contrast-enhanced sagittal CT image showing cranio-caudal extension of the spleen.

**Fig. 3:**
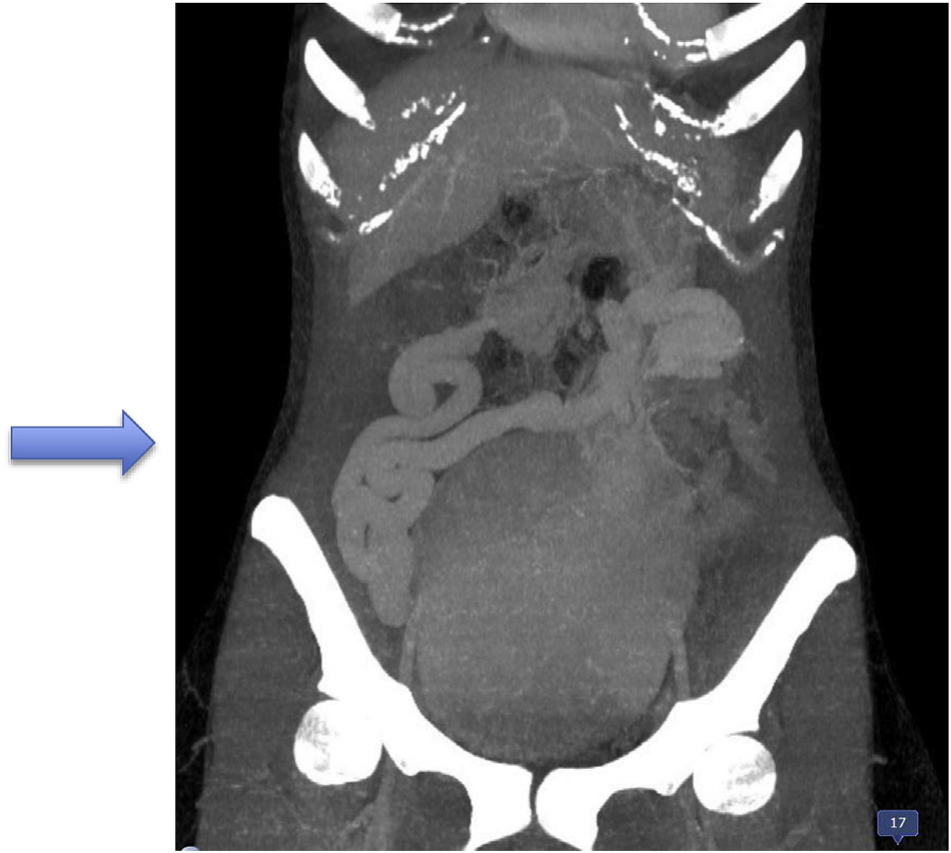
Contrast-enhanced coronal CT image, MPR reconstructions, shows the pelvic location of the megalic spleen and huge ectasic veins.

**Fig. 4:**
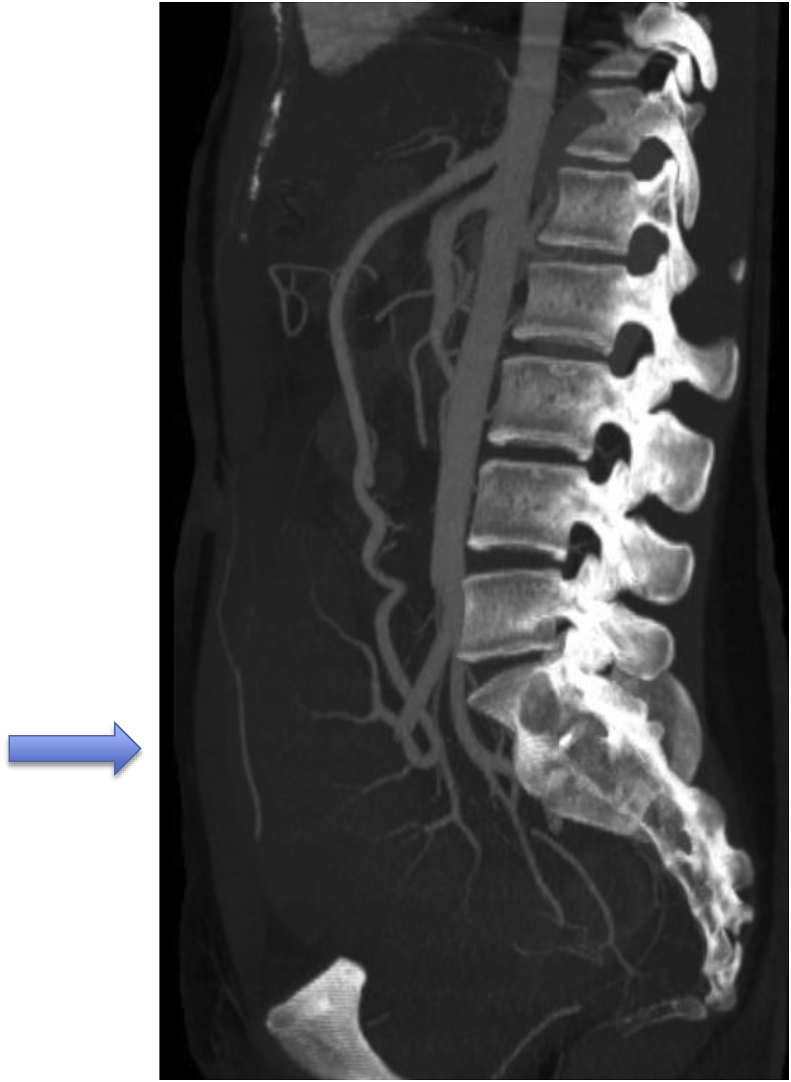
Axial contrast-enhanced CT image, MPR reconstructions, shows the splenic hilum.

She was investigated with a new CT-scan of abdomen and pelvis that showed a multi-infarcted pelvic splenomegaly (max diameter 13,5 cm), compressing the womb and the bladder, and a typical “whirl sign” of the splenic vascular pedicle (Fig. 1, Fig. 2). No radiological signs of portal hypertension were found, but was reported huge ectasia of the splenic veins and collateral tributaries (Fig. 3). Angio-CT reconstruction was performed to study in details the vascularization of the spleen (Fig. 4).

She underwent hematological evaluation revealing normocromic normocytic anemia and piastrinopenia compatible with the multi-infarcted spleen. Also an Helicobater Pylori gastritis was found and treated by eradication therapy.

A physical examination of the abdomen revealed a tender pelvic mass, changing position with the variation of the patient decubitus.

The patient was candidated to a laparoscopic elective splenectomy due to her symptoms, the spleen dimension, the risk of splenic infarctions and potential obstacle to a future pregnancy. She received a prophylaxis therapy with triple vaccine against Haemophilus Influenzae, Pneumococcus and Meningococcus three weeks before surgery.

An explorative laparoscopy confirmed the presence of a bulky multi-infarcted spleen completely obstructing the pelvis. The vascular pedicle was composed of a big artery originating as usual from the celiac trunk and some enormous veins draining into the superior mesenteric branch. Because of the unusual position and weight of the spleen and its vessels, also the tail of the pancreas was innaturally tractioned medially and inferiorly towards the lower abdomen. Furthermore the pancreas appeared to be in abnormal position, resulting anterior to the mesocolon and intraperitoneally located.

The *Sustentaculum lienis* (freno-colic, colic and freno-splenic ligaments) was under-developed; the splenic pedicle was twirled around its long axis several times and the tail of pancreas was partially involved in the torsion without any chance of derotation. To avoid the risk of complications, like vein thrombosis or bleeding from the ectasic tributaries of the splenic vein, we decided to dissect the splenic pedicle in proximity of the tail of pancreas preserving the pancreatic vessels.

**Fig. 5:**
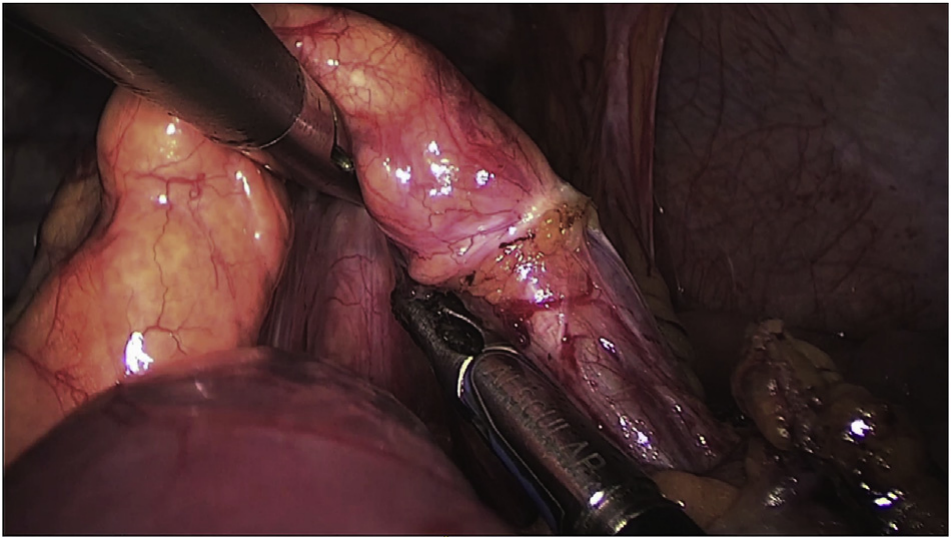
Intraoperative view showing the splenic vascular pedicle directly attached to the pancreatic tail.

**Fig. 6:**
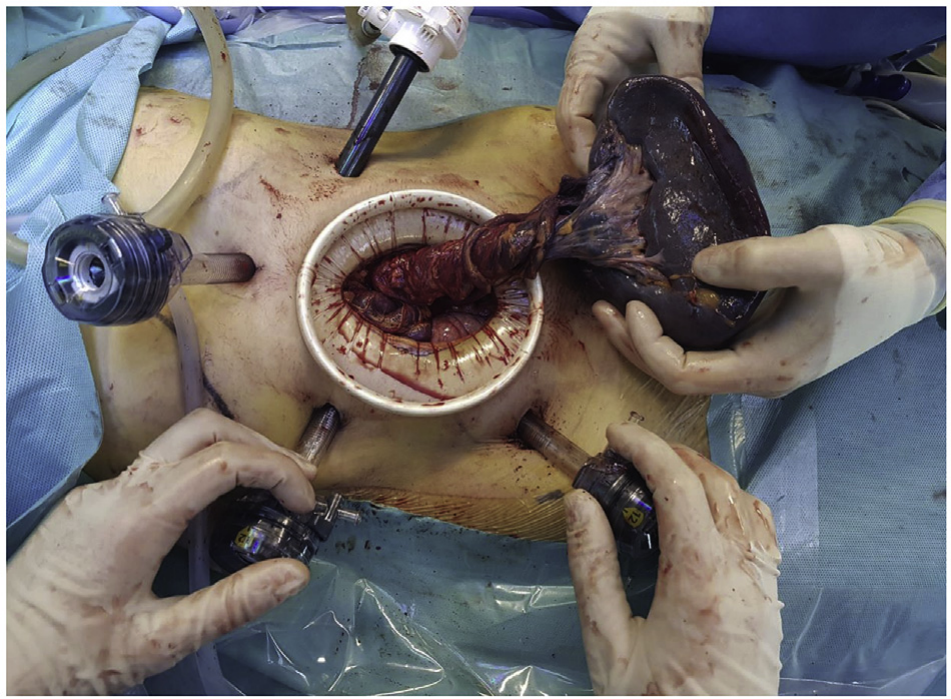
Intraoperative view showing an enlarged spleen with a long a twisted vascular pedicle.

A laparoscopic dissection and ligature of the splenic artery was performed, obtaining a volume reduction of the spleen. We than continued with the ligation of some ectasic splenic veins and finally, we extracted the specimen from a 5cm periumbilical incision and completed the separation of the splenic pedicle from the pancreatic tail (Fig. 5, Fig. 6).

After splenectomy, the tail of pancreas was left in its innatural position, above the left colic flexure. A peripancreatic drainage was placed. (Supplemental Digital Content: Video)

Supplementary video related to this article can be found at https://doi.org/10.1016/j.amsu.2019.12.001.

The following is the supplementary data related to this article:

Wandering_spleen Wandering_spleen

In order to reduce the risk of postoperative pancreatitis, we started intraoperative octreotide, extended during the all hospitalization.

The post-operative recovery of the patient was uneventful and she was discharged after 6 days.

Laboratory investigations showed a postoperative stability of hemoglobin level and a progressive increased platelet count.

Given the risk of thrombotic events, prophylaxis with subcutaneous low molecular weight heparin (LMWH) was started 24 hours before undergoing surgery and continued for 1 month.

Histopathological examination revealed a spleen measuring 15 cm (maximum diameter), weighing 576 g, with multiple twist of the pedicle, which was revealed to be 45 cm long. The microscopic examination showed multiple hemorrhagic infarctions.

## 3 Discussion and conclusions

Early diagnosis of wandering spleen is very important, as it turns in the chance to preserve the organ function, avoiding splenectomy and all its risks, of which the major is represented by the Overwhelming Postsplenectomy Sepsis (OPSS).

In fact, splenopexy is the first line treatment in asymptomatic patients or in the symptomatic ones, whose spleen is shown to be non-infarcted, normal sized, and without signs of hypersplenism. Detorsion and splenopexy may be considered a surgical option even in emergency settings, when there is no evidence of infarction, thrombosis or hypersplenism; while splenectomy must be reserved to complicated forms of WS, when necrosis of the parenchyma is present.

In the past, many surgeons used to recommend non-operative management in asymptomatic patients, but a delayed diagnosis or treatment turned out to be related to many more life-threatening complications, such as torsion of vascular pedicle, gangrene of the spleen, severe bleeding from esophageal varices, intestinal obstruction, gastric or intestinal gangrene, and acute pancreatitis. Nowadays, there are still no clear data about the potential risks of a trauma involving an unprotected and abnormally located pelvic spleen. Therefore, conservative management is not recommended anymore, even if the patients is asymptomatic (the treatment of asymptomatic elderly patients is still under discussion).

According to the scientific community, in case surgery should be performed, laparoscopic approach is the preferred one; there is, anyway, no total agreement about the best laparoscopic technique, whether splenopexy or splenectomy. Since 2000, splenopexy is the first line treatment in wandering spleen. This technique, which consists in the fixation of the spleen in its normal anatomic site, is the treatment of choice in asymptomatic patients or in the symptomatic ones, of whom the spleen is shown to be non-infarcted, normal sized, and has no signs of hypersplenism. Laparoscopic splenopexy has many advantages over open splenopexy or splenectomy, including preserving splenic function, minimal postoperative pain, early discharge from the hospital, and rapid recovery. Splenopexy has been increasingly used especially in the paediatric population, because of the immunity role of this organ during the childhood. Contraindications to laparoscopic approach are represented by coagulopathies and severe portal hypertension. Therefore, spleen size must be taken in consideration in case of a planned laparoscopic approach, because it has a direct relation with an increasing risk of conversion to open surgery.

We could not find any reports about the prophylactic embolization of the splenic artery, but we suggest a possible role of this technique both in the conservative management of the WS and the operative one. In fact the embolization of the splenic artery 24–48 hours before splenectomy, can be considered in case of a laparoscopic management, because it could reduce the risk of intraoperative bleeding, the volume of the organ and the dimension of the ectasic veins.

In conclusion, wandering spleen is a rare clinical condition that should ideally be diagnosed early, before severe symptoms could occur and treated conservatively with an elective laparoscopic splenopexis. When not possible, laparoscopic splenectomy is effective and safe. Studies on prophylactic embolization of the splenic artery in wandering spleen should be performed.

## Declarations

### Ethics approval and consent to participate

Written informed consent was obtained from the patient for publication of this Case report and any accompanying images. A copy of the written consent is available for review by the Editor of this journal. This case report study was carried out respecting the Declaration of Helsinki in its current version. Ethical approval: not applicable.

### Consent for publication

Written and informed consent was taken from the patient for publication of this case report and any accompanying images. A copy of the written consent is available for review by the Editor-in-Chief of this journal.

### Availability of data and materials

Not applicable.

### Funding

No outside support was provided for the report.

### Authors’ contributions

All authors were involved in the preparation of this manuscript. FC, CB and PDA collected the data, and wrote the manuscript. FC and MC performed the operation and designed the study. FC, GS and DF summarized the data and revised the manuscript. MC, GS and DF made substantial contribution to the study design and revised the manuscript. All authors read and approved the final manuscript.

## Trial registry number

Not applicable.

## Guarantor

Dr Francesco Colombo.

## Consent

Written and informed consent was taken from the patient for publication of this case report and any accompanying images. A copy of the written consent is available for review by the Editor-in-Chief of this journal.

## Provenance and peer review

Not commissioned, externally peer reviewed.

## Declaration of competing interest

The authors declare that they have no competing interests.

## List of Abbreviations

WS: wandering spleen
CT: computed tomography
LMWH: low molecular weight heparin
OPSS: overwhelming postsplenectomy sepsis

## Appendix A Supplementary data

SCARE Checklist pag 1 SCARE Checklist pag 1

SCARE Checklist pag 2 SCARE Checklist pag 2

SCARE Checklist pag 3
