# Supplementary material for: Torsion of wandering spleen involving the pancreatic tail
Source: Ann Med Surg (Lond). 2019 Dec 20;50:10–3. doi: 10.1016/j.amsu.2019.12.001 (PMC6994766; doi:10.1016/j.amsu.2019.12.001)
Supplement: SCARE Checklist pag 1 [file mmc2.pdf]

SCARE Checklist

Topic	Item	Checklist item description	Page Number
Title	1	The words "case report" and the area of focus should appear in the title (e.g. presentation, diagnosis, surgical technique or device or outcome).	PAG 1
Key Words	2	3 to 6 key words that identify areas covered in this case report (include "case report" as one of the keywords).	PAG 1
Abstract	3a	Introduction—What is unique or educational about the case? What does it add to the surgical literature? Why is this important?	PAG 1
	3b	The patient's main concerns and important clinical findings.	
	3c	The main diagnoses, therapeutics interventions, and outcomes.	
	3d	Conclusion — what are the "take-away" lessons from this case?	
Introduction	4	A summary of why this case is unique or educational with reference to the relevant surgical literature and current standard of care (with references, 1-2 paragraphs). Nature of the institution in which the patient was managed; academic, community or private practice setting?	PAG 2
Patient Information	5a	De-identified demographic and other patient specific information including age, sex, ethnicity, occupation and other useful pertinent information e.g. BMI and hand dominance.	PAG 3
	5b	Presentation including presenting complaint and symptoms of the patient as well as the mode of presentation e.g. brought in by ambulance or walked into Emergency room or referred by family physician.	
	5c	Past medical and surgical history and relevant outcomes from interventions	
	5d	Drug history, family history including any relevant genetic information, and psychosocial history including smoking status and where relevant accommodation type, walking aids, etc.	
Clinical Findings	6	Describe the relevant physical examination and other significant clinical findings (include clinical photographs where relevant and where consent has been given).	PAG 3
