# Supplementary material for: Torsion of wandering spleen involving the pancreatic tail
Source: Ann Med Surg (Lond). 2019 Dec 20;50:10–3. doi: 10.1016/j.amsu.2019.12.001 (PMC6994766; doi:10.1016/j.amsu.2019.12.001)
Supplement: SCARE Checklist pag 2 [file mmc3.pdf]

Timeline	7	Inclusion of data which allows readers to establish the sequence and order of events in the patient's history and presentation (using a table or figure if this helps). Delay from presentation to intervention should be reported.	PAG 3
Diagnostic Assessment	8a	Diagnostic methods (physical exam, laboratory testing, radiological imaging, histopathology etc).	PAG 3
	8b	Diagnostic challenges (access, financial, cultural).	
	8c	Diagnostic reasoning including other diagnoses considered	
	8d	Prognostic characteristics when applicable (e.g. tumour staging). Include relevant radiological or histopathological images in this section (the latter may sometimes be better placed in section 9).	
Therapeutic Intervention	9a	Pre-intervention considerations e.g. Patient optimisation: measures taken prior to surgery or other intervention e.g. treating hypothermia/hypovolaemia/hypotension in a burns patient, ICU care for sepsis, dealing with anticoagulation/other medications, etc	PAG 3-4
	9b	Types of intervention(s) deployed and reasoning behind treatment offered (pharmacologic, surgical, physiotherapy, psychological, preventive) and concurrent treatments (antibiotics, analgesia, anti-emetics, nil by mouth, VTE prophylaxis, etc). Medical devices should have manufacturer and model specifically mentioned.	
	9c	Peri-intervention considerations - administration of intervention (what, where, when and how was it done, including for surgery; anaesthesia, patient position, use of tourniquet and other relevant equipment, prep used, sutures, devices, surgical stage (1 or 2 stage, etc). Pharmacological therapies should include formulation, dosage, strength, route, duration, etc).	
	9d	Who performed the procedure - operator experience (position on the learning curve for the technique if established, specialisation and prior relevant training).	
	9e	Any changes in the interventions with rationale. Include intra-operative photographs and/or video or relevant histopathology in this section. Degree of novelty for a surgical technique/device should be mentioned e.g. "first in-human".	
	9f	Post-intervention considerations e.g. post-operative instructions and place of care.	
Follow-up and Outcomes	10a	Clinician assessed and patient-reported outcomes (when appropriate) should be stated with inclusion of the time periods at which assessed. Relevant photographs/radiological images should provided e.g. 12 month follow-up.	PAG 4
