# Supplementary material for: Torsion of wandering spleen involving the pancreatic tail
Source: Ann Med Surg (Lond). 2019 Dec 20;50:10–3. doi: 10.1016/j.amsu.2019.12.001 (PMC6994766; doi:10.1016/j.amsu.2019.12.001)
Supplement: Supplementary file 4 — SCARE Checklist pag 3 [file mmc4.pdf]

	10b	Important follow-up measures - diagnostic and other test results. Future surveillance requirements - e.g. imaging surveillance of endovascular aneurysm repair (EVAR) or clinical exam/ultrasound of regional lymph nodes for skin cancer.	/
	10c	Where relevant - intervention adherence and tolerability (how was this assessed).	PAG 4
	10d	Complications and adverse or unanticipated events. Described in detail and ideally categorised in accordance with the Clavien-Dindo Classification. How they were prevented, diagnosed and managed. Blood loss, operative time, wound complications, re-exploration/revision surgery, 30-day post-op and long-term morbidity/mortality may need to be specified.	
**Discussion**	11a	Strengths, weaknesses and limitations in your approach to this case. For new techniques or implants - contraindications and alternatives, potential risks and possible complications if applied to a larger population. If relevant, has the case been reported to the relevant national agency or pharmaceutical company (e.g. an adverse reaction to a device).	PAG 4-5
	11b	Discussion of the relevant literature, implications for clinical practice guidelines and any relevant hypothesis generation.	
	11c	The rationale for your conclusions.	
	11d	The primary "take-away" lessons from this case report.	
**Patient Perspective**	12	When appropriate the patient should share their perspective on the treatments they received.	/
**Informed Consent**	13	Did the patient give informed consent for publication? Please provide if requested by the journal/editor. If not given by the patient, explain why e.g. death of patient and consent provided by next of kin or if patient/family untraceable then document efforts to trace them and who within the hospital is acting as a guarantor of the case report.	PAG 6
**Additional Information**	14	Conflicts of Interest, sources of funding, institutional review board or ethical committee approval where required.	PAG 6
